# Adoption of MRI-guided Prostate Cancer Diagnostics and Surgical Outcomes: A Prospective Multicenter Registry Study

**DOI:** 10.1016/j.euros.2026.05.021

**Published:** 2026-07-10

**Authors:** Angelos Tasios, Nicolas Arnold, Raphael Röthlisberger, Stephen Wyler, Daniel Phat Nguyen, Dominik Abt, Agostino Mattei, Räto Strebel, George Thalmann, Laila Schneidewind, Beat Roth, Nicola Giudici, Swiss Urology Registry Collaborative Group, Karim Kellou, Thomas Sautter, Wolfgang Schäfer, Anja Rieger, Simone Brunschweiler, Thomas Tawadros, Thomas Sautter, Aron Cohen, Daniel Nguyen, Gautier Müllhaupt, Stefan Preusser, Ilaria Lucca, Beat Roth, Thomas Luginbühl, Roland Seiler, Astrid Bergundthal, Olivier Ischer, Jérôme Chaptinel, Massimo Valerio, Räto Strebel, Daniel Engeler, Daniel Eberli, Tobias Zellweger, Agostino Mattei, Hubert John, Stephen Wyler, Denise Bundi, Julien Schwartz, Stephan Bauer

**Affiliations:** 1 Hôpital de Nyon, Nyon, Switzerland; 2 Uroclinic, Wetzikon, Switzerland; 3 Spitalzentrum Oberwallis, Visp, Switzerland; 4 Limmattalspital, Schlieren, Switzerland; 5 Bellevue Urology, Zürich, Switzerland; 6 Hôpital Riviera-Chablais, Rennaz, Switzerland; 7 Uroclinic, Pfäffikon, Switzerland; 8 Urocare AG, Küsnacht, Switzerland; 9 Hôpital Neuchâtelois, Neuchâtel, Switzerland; 10 Spital Thun (SpitalSTS AG), Thun, Switzerland; 11 Kantonsspital Schaffhausen, Schaffhausen, Switzerland; 12 CHUV - Centre Hospitalier Universitaire Vaudois, Lausanne, Switzerland; 13 Inselspital, Bern, Switzerland; 14 Spital Uster, Uster, Switzerland; 15 Spitalzentrum Biel, Biel, Switzerland; 16 Hirslanden Klinik Stephanshorn, St. Gallen, Switzerland; 17 Clinique de la Source, Lausanne, Switzerland; 18 Hôpitaux Universitaires de Genève (HUG), Genève, Switzerland; 19 Kantonsspital Graubünden, Chur, Switzerland; 20 Kantonsspital St. Gallen (KSSG), St. Gallen, Switzerland; 21 Universitätsspital Zürich (USZ), Zürich, Switzerland; 22 St. Claraspital, Basel, Switzerland; 23 Luzerner Kantonsspital (LUKS), Luzern, Switzerland; 24 Kantonsspital Winterthur (KSW), Winterthur, Switzerland; 25 Kantonsspital Aarau, Aarau, Switzerland; 26 Uroviva, Bülach, Switzerland; 27 Hirslanden Clinique Cecil, Lausanne, Switzerland; 28 Hirslanden Zentrum für Urologie (ZfU), Zürich, Switzerland; a Inselspital, Department of Urology, Bern, Switzerland; b Department of Urology, Cantonal Hospital Aarau, Aarau, Switzerland; c Department of Urology, Réseau Hospitalier Neuchâtelois, Neuchâtel, Switzerland; d Department of Urology, Spitalzentrum Biel, Centre Hospitalier Bienne, Biel, Switzerland; e Department of Urology, University of Lucerne, Luzerner Kantonsspital, Lucerne, Switzerland; f Department of Urology, Cantonal Hospital of Graubünden, Chur, Switzerland

**Keywords:** Prostate MRI, Prostate biopsy, Fusion biopsy, Template biopsy, Radical prostatectomy

## Abstract

**Background:**

Adoption of magnetic resonance imaging (MRI)–guided prostate biopsy across different health care settings remains variable, and its implementation, following guideline endorsement, has not been fully characterized, particularly with respect to temporal trends and downstream surgical outcomes.

**Design, setting, and participants:**

We analyzed biopsy techniques and postoperative outcomes among men undergoing radical prostatectomy (2020–2025), using the prospective national SWISS urology prostatectomy registry.

**Outcome measurements and statistical analysis:**

Temporal trends in MRI-guided versus non–MRI-guided biopsy were compared across university, regional, and local/private hospitals. Multivariable logistic regression models were used to evaluate associations between biopsy technique and pathological and perioperative outcomes.

**Results and limitations:**

A total of 7687 men from 29 centers were included. A rapid transition from non–MRI-guided to MRI-guided biopsy was observed over the study period. University hospitals were early adopters of MRI-guided biopsy, with use approaching 85% by 2020 and achieving complete adoption by 2024. Regional and local/private hospitals showed a more gradual increase, with MRI-guided biopsy rising from 44% in 2020 to 88% by early 2025. In multivariable analyses, MRI-guided biopsy was associated with lower risk of understaging (odds ratio [OR] 0.64; 95% confidence interval [CI] 0.57–0.72; *p* < 0.001) and positive surgical margins (OR 0.80; 95% CI 0.70–0.92; *p* = 0.001), and was not associated with increased perioperative complications or International Society of Urological Pathology grade upgrading. Limitations include residual confounding and the selection of surgically treated patients.

**Conclusions:**

MRI-guided biopsy rapidly became the dominant diagnostic approach, with academic centers leading adoption and other care settings converging thereafter, highlighting the important role of academic centers in implementing guideline-recommended diagnostics.

## Take Home Message

Magnetic resonance imaging (MRI)–guided prostate biopsy has rapidly become a standard practice, with academic centers leading its adoption. Its use is associated with improved staging accuracy and lower rates of positive surgical margins without an increase in perioperative risk, supporting the role of an MRI-informed diagnostic pathway as a standard step before surgery.

ADVANCING PRACTICE **What does this study add?**

This nationwide registry study provides real-world evidence on the rapid implementation of magnetic resonance imaging (MRI)–guided prostate biopsy across different health care settings, following guideline endorsement. It demonstrates that academic centers act as early adopters, with subsequent convergence across regional and local institutions. Importantly, MRI-guided biopsy was independently associated with improved staging accuracy and lower rates of positive surgical margins without increasing perioperative risk. These findings highlight that the timely adoption of MRI-based diagnostics translates into measurable improvements in surgical quality at a population level.

**Clinical Relevance**

These findings support the routine integration of MRI into the prostate cancer diagnostic pathway, as MRI-guided biopsy was associated with improved pathological staging accuracy and reduced positive surgical margins at radical prostatectomy. However, MRI may not be required for all men and could potentially be omitted in those at low risk of clinically significant prostate cancer when PSA density and validated risk calculators indicate a low likelihood of disease. Conversely, in men with a very high probability of advanced cancer (e.g., PSA >20–50 ng/mL or clinical stage cT3–4), MRI before biopsy may have limited impact on the diagnostic pathway, suggesting that a risk-adapted approach to MRI use may optimize resource utilization without compromising care. Associate Editor: Roderick C.N. van den Bergh, MD PhD.

**Patient Summary**

In this study, we looked at how prostate biopsy techniques have changed over time and how this affects surgery outcomes. We found that magnetic resonance imaging–guided biopsy is now widely used and helps better understand the extent of the cancer before surgery. This leads to more accurate treatment and better surgical results without increasing risks for patients.

## 1 Introduction

Prostate cancer (PCa) is the most commonly diagnosed tumor among men in Europe and remains a major contributor to cancer-related morbidity and mortality [1]. Although prostate-specific antigen (PSA) testing supports early detection, prostate biopsy remains an essential component of the diagnostic workup, providing definitive histopathological confirmation, International Society of Urological Pathology (ISUP) grading, and guidance for treatment planning [2], [3]. Therefore, the accuracy of the initial biopsy is critical, as misclassification may lead to significant clinical consequences, including overtreatment or delayed intervention for clinically significant disease [4], [5], [6].

For decades, systematic transrectal ultrasound-guided biopsy represented the diagnostic standard. Magnetic resonance imaging (MRI)–targeted biopsy, whether cognitive, MRI-ultrasound fusion, or in bore, has demonstrated superior detection of clinically significant PCa, reduced oversampling of indolent tumors, and lower rates of pathological upgrading at radical prostatectomy [7], [8], [9], [10]. A previous Swiss study combining single-center biopsy data with national cancer registry data further showed that adoption of multiparametric MRI (mpMRI)–guided biopsy was associated with fewer negative biopsies and a shift toward higher-risk PCa detection [11]. The European Association of Urology guidelines, in their 2020 update, strongly recommended the use of prebiopsy mpMRI with subsequent targeted biopsy if positive. This update shifted from a weak recommendation in 2019 to a strong one with Level 1A evidence, fundamentally incorporating MRI-targeted and fusion biopsies into the standard diagnostic pathway [2], [12].

Despite strong evidence supporting MRI-targeted diagnostic strategies, their uptake varies across health care systems and is influenced by institutional resources, imaging infrastructure, and guideline implementation [13], [14]. Academic centers, regional hospitals, and local institutions all play a role in the implementation of evidence-based innovations; however, differences in infrastructure, access to expertise, and organizational capacity may influence the timing and extent of adoption [15]. These observed variations in implementation across care settings primarily prompted the research question addressed in this study, which used prospectively collected data from 29 centers in the Swiss Urology Prostatectomy Registry, to examine 5-yr trends (2020–2025) in MRI-guided versus non–MRI-guided prostate biopsy across university, regional, and local/private hospitals and assess associations with pathological and perioperative outcomes after radical prostatectomy. Although we acknowledge that a definitive causal relationship cannot be established because of the study design, the aim of our study was to examine whether the biopsy technique is associated with pathological and perioperative outcomes after radical prostatectomy.

## 2 Materials and methods

### 2.1 Data source

This study uses data from the national, prospective registry of SWISS UROLOGY, a quality-monitoring initiative launched in 2020 that also encompasses procedure-specific registries, such as cystectomy, prostatectomy, and nephrectomy, with the overarching aim of assessing outcomes across Switzerland. The structure, data quality, and validity of the registries have been described previously, including analyses demonstrating their applicability for outcome research [16], [17]. Participation in the registry increased over time. In 2020, a total of 473 radical prostatectomies were recorded, corresponding to 14% of the 3284 procedures performed nationwide. This proportion increased to 41% in 2021, 50% in 2022, and remained stable at around 50% in 2023 and 2024, reflecting a progressive expansion and subsequent stabilization of registry coverage over the study period. Since July 1, 2025, participation has been mandatory for all certified urological training centers, expanding national coverage to roughly 90%. Data are entered into a standardized electronic platform (Adjumed, CH-8055 Zürich, Switzerland) using a prospective, two-step data capture process. Operative and perioperative variables are recorded at the time of surgery by the responsible surgeon, whereas follow-up data and postoperative outcomes are updated periodically by a dedicated study nurse as part of routine registry follow-up. All data are pseudonymized before central aggregation. The registry systematically documents patient-, tumor-, treatment-, and outcome-related variables, with follow-up at 3, 12, and 24 mo to capture disease course, complications, adjuvant management, and survival. Data quality is ensured through automated plausibility checks and periodic audits coordinated by SWISS UROLOGY. The study was approved by the Cantonal Ethics Committee of Zurich (BASEC-Nr. Req-2017-00283).

### 2.2 Outcomes and exposure definition

The primary exposure of interest was biopsy technique, categorized as: MRI-guided biopsy, non–MRI-guided biopsy, and TUR-P/Other. The TUR-P/Other category was excluded from inferential analyses because its incidence was low and the procedures are outside the conceptual scope of this study, which focuses on comparing contemporary prostate biopsy techniques. The primary end point was descriptive, examining temporal trends in biopsy technique use between 2020 and 2025 across different hospital settings. University hospitals represent academic tertiary referral centers (five in total in Switzerland). Regional hospitals correspond to non-university public secondary care centers, typically the main cantonal hospitals serving cantons without a university hospital. Local/private institutions include smaller non-academic public hospitals and private clinics. Secondary end points involved multivariable modeling to assess whether biopsy technique was independently associated with postoperative pathological and perioperative outcomes following radical prostatectomy. These included the following: ISUP upgrading, defined as a higher postoperative ISUP grade compared with the preoperative ISUP grade biopsy; clinical local understaging, defined as cT-stage < pT-stage; positive surgical margins (PSM); and intraoperative complications recorded at the time of prostatectomy.

### 2.3 Missing data

Missing data were low across all covariates included in the adjusted models, with <3% missing for each variable (age, American Society of Anesthesiologists [ASA] score, clinical T-stage, preoperative PSA, preoperative ISUP grade, prostate volume, surgical technique, and hospital type). In light of this minimal missingness, multivariable analyses were conducted using a complete-case approach, leading to the exclusion of 311 of the 7687 (4.0%) patients.

### 2.4 Statistical analysis

Baseline characteristics were compared across biopsy techniques. Continuous variables were summarized as medians with interquartile ranges (IQRs), and categorical variables as frequencies and percentages. Group comparisons were performed using the Wilcoxon rank-sum test for continuous variables and the chi-square test for categorical variables. To evaluate changes in biopsy practice over time, the proportion of MRI-guided versus non–MRI-guided biopsy was assessed using chi-square tests, comparing the beginning and end of the study period separately within university hospitals and non-university hospitals. To evaluate the association between biopsy technique and each outcome, multivariable logistic regression models were fitted, with biopsy technique as the main exposure. Patients diagnosed via TUR-P or other nonstandard diagnostic pathways were excluded from all inferential analyses. A complete-case approach was used for multivariable modeling. Models were adjusted for age, preoperative PSA, preoperative ISUP grade, clinical T-stage, prostate volume, hospital type (other vs university hospitals), year of biopsy, ASA score, and surgical technique (laparoscopic/robotic vs open/converted). For analyses of ISUP upgrading and clinical understaging, the corresponding preoperative variables were omitted, as they form part of the outcome definition. Results are presented as adjusted odds ratios (ORs) with 95% confidence intervals (CIs) and two-sided *p* values, with statistical significance defined as *p* < 0.05.

All analyses were performed in R version 4.4.1 (R Foundation for Statistical Computing, Vienna, Austria).

## 3 Results

### 3.1 Baseline characteristics

Data from 7687 men who underwent radical prostatectomy were included in the analysis. Baseline characteristics stratified by biopsy technique are presented in Table 1, with corresponding data for patients diagnosed via TUR-P and nonstandard diagnostic pathway shown in Supplementary Table 2. Patient age, body mass index, and ASA score were similar across biopsy groups. Preoperative PSA levels were slightly higher in the non–MRI-guided group (median 7.5 ng/ml [IQR 5.2–11.5] vs 7.1 ng/ml [IQR 5.0–10.5]; *p* = 0.001), whereas prostate volume was comparable between groups. The distribution of D’Amico risk categories differed between groups, with a higher proportion of patients with intermediate-risk disease in the non–MRI-guided group and a higher proportion of patients with high-risk disease in the MRI-guided group (intermediate risk 65% vs 57%; high risk 30% vs 36%, *p* < 0.001). As expected, MRI-guided biopsy was associated with a higher number of biopsy cores taken (median 15 cores [IQR 12–19] vs 12 cores [IQR 12–15]; *p* < 0.001), whereas the number of positive cores was similar. Operative characteristics were broadly comparable, although small differences were observed, with longer operative time (median 192 min [IQR 150–240] vs 185 min [IQR 150–224]; *p* < 0.001), higher lymph node dissection rates (77% vs 73%; *p* = 0.002), and lower rates of nerve-sparing procedures (25% vs 30%; *p* < 0.001) in the MRI-guided compared with the non–MRI-guided group, respectively. With respect to pathological and postoperative outcomes, crude rates of PSM were lower in the MRI-guided group (22% vs 28%; *p* < 0.001), whereas the distribution of postoperative ISUP grades and intraoperative complication rates were similar. Overall postoperative complication rates differed between groups, with higher rates in the MRI-guided compared with the non–MRI-guided group (13% vs 9.3%; *p* < 0.001), although absolute event rates remained low.

**Table 1:** Baseline, operative, and pathologic characteristics of each biopsy technique

Variable	MRI-guided biopsy, *N* = 5432	Non–MRI-guided biopsy, *N* = 1827	*p* value
Institution and demographics			
Hospital type, *n* (%)			
University hospital	668 (12)	28 (1.5)	<0.001
Regional hospital	1792 (33)	627 (34)	
Local/private hospital	2972 (55)	1172 (64)	
Age (yr), median (IQR)	67 (61–71)	67 (62–71)	0.8
BMI (kg/m^2^), median (IQR)	26.3 (24.2–28.7)	26.2 (24.2–28.7)	1
ASA score, *n* (%)			
I–II	3477 (64)	1268 (69)	0.16
≥III	1295 (24)	417 (23)	
Preoperative characteristics			
Preoperative PSA (ng/ml), median (IQR)	7.1 (5.0–10.5)	7.5 (5.2–11.5)	0.001
D’Amico risk groups, *n* (%)			
Low risk	299 (5.5)	86 (4.7)	<0.001
Intermediate risk	3094 (57)	1181 (65)	
High risk	1968 (36)	541 (30)	
Prostate volume (ml), median (IQR)	41 (31–55)	40 (30–54)	0.091
Number of biopsy cores taken, median (IQR)	15 (12–19)	12 (12–15)	<0.001
Number of positive biopsy cores, median (IQR)	5 (3–8)	5 (3–8)	0.8
Operative characteristics			
Surgical technique, *n* (%)			
Robotic/laparoscopic-assisted approach	4939 (91)	1636 (90)	0.032
Open approach/converted	445 (8.2)	163 (8.9)	
Pelvic lymph node dissection	4184 (77)	1341 (73)	0.002
Nerve sparing performed	1343 (25)	548 (30)	<0.001
Estimated blood loss (ml), median (IQR)	200 (100–300)	200 (113–300)	0.001
Operative time (min), median (IQR)	192 (150–240)	185 (150–224)	<0.001
Postoperative and pathologic outcomes			
Pathologic T stage, *n* (%)			
pT2	3514 (65)	1133 (62)	0.032
≥pT3	1882 (35)	690 (38)	
Postoperative ISUP, *n* (%)			
1–2	3177 (59)	1087 (60)	0.2
3–5	2207 (41%)	736 (40)	
Positive surgical margin, *n* (%)	1211 (22)	511 (28)	<0.001
Lymph nodes examined, median (IQR)	11 (3–18)	10 (1–16)	<0.001
Any intraoperative complication, *n* (%)	77 (1.4)	25 (1.4)	1
Any postoperative complication (30 d), *n* (%)	694 (13)	171 (9.3)	<0.001
Clavien-Dindo grade, *n* (%)			
I–II	258 (4.8)	72 (3.9)	0.16
≥III	79 (1.5)	32 (1.8)	

ASA = American Society of Anesthesiologists; BMI = body mass index; IQR = interquartile range; ISUP = International Society of Urological Pathology; MRI = magnetic resonance imaging; PSA = prostate-specific antigen.

Transurethral resection of the prostate (TURP)/other cases (*n* = 356) are presented separately in Supplementary Table 2.

### 3.2 Perioperative and pathologic characteristics by biopsy technique

**Fig. 1:**
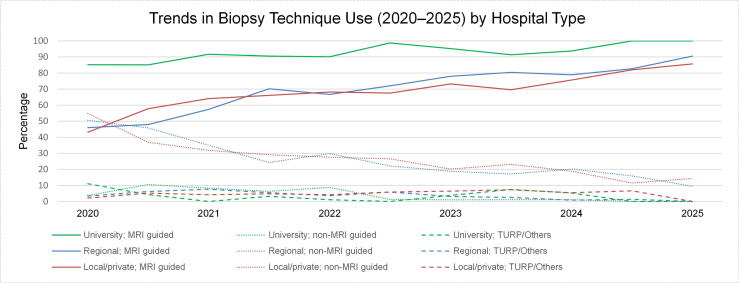
Trends in biopsy technique use by hospital type between 2020 and 2025. **Abbreviations:** MRI = magnetic resonance imaging; TURP = transurethral resection of the prostate.

A clear nationwide transition from non–MRI- to MRI-guided biopsy was observed between 2020 and 2025 (Fig. 1; Supplementary Table 3). In nonuniversity hospitals, MRI-guided biopsy increased from 44% in 2020 to 88% in 2025 (*p* < 0.001). University hospitals showed much earlier and more complete adoption, with MRI-guided biopsy already exceeding 85% in 2020 and reaching 100% by 2024 (*p* < 0.001). Regional and local/private hospitals showed a more gradual but consistent increase in MRI-guided biopsy use, converging toward similarly high adoption rates by 2025. Non–MRI-guided biopsy steadily declined across all settings and became rare in later years, whereas TUR-P/other procedures remained infrequent and stable throughout the study period. Overall, MRI-guided biopsy emerged as the predominant diagnostic modality, with academic centers acting as early adopters and regional and local/private institutions progressively aligning over time.

### 3.3 Adjusted pathologic and perioperative outcomes

Of the 7687 patients included in the descriptive analyses, 356 diagnosed via TUR-P or other nonstandard pathways and 311 with missing data were excluded, resulting in a complete-case cohort of 7020 patients for adjusted analyses. Table 2 presents the risk-adjusted association between biopsy technique and key postprostatectomy outcomes. Biopsy technique was not associated with ISUP upgrading (OR 1.10; 95% CI 0.95–1.27; *p* = 0.2). MRI-guided biopsy was associated with lower risk of clinical understaging (OR 0.64; 95% CI 0.57–0.72; *p* < 0.001) and PSM (OR 0.80; 95% CI 0.70–0.92; *p* = 0.001). No association was observed between biopsy technique and overall postoperative complications (OR 1.03; 95% CI 0.84–1.27; *p* = 0.8). The complete multivariable regression analyses, including all covariates used for adjustment, are shown in the Supplementary Tables 4–7.

**Table 2:** Multivariable logistic regression evaluating biopsy technique as a predictor of postprostatectomy outcomes

Outcome	Adjusted OR for MRI-guided biopsy	95% CI	*p* value
ISUP upgrading (RP vs biopsy)	1.10	0.95–1.27	0.2
Understaging (cT < pT)	0.64	0.57–0.72	<0.001
Positive surgical margins	0.80	0.70–0.92	0.001
Overall postoperative complications	1.03	0.84–1.27	0.8

CI = confidence interval; cT = clinical T Stage; pT = pathological T Stage; ISUP = International Society of Urological Pathology; MRI = magnetic resonance imaging; OR = odds ratio; RP = Radical Prostatectomy.

## 4 Discussion

In this nationwide analysis, we evaluated temporal trends in the adoption of guideline-recommended prostate biopsy techniques and observed a rapid, near-complete transition from non–MRI-guided to MRI-guided biopsy between 2020 and 2025, with marked differences in the pace of implementation across care settings. University hospitals implemented MRI-guided biopsy earlier and more comprehensively, whereas regional hospitals and local/private institutions followed a more gradual adoption trajectory. These findings align with broader observations in health care innovation research, suggesting that institutional characteristics, such as infrastructure, access to specialized expertise, and organizational capacity, play a decisive role in determining how rapidly new diagnostic technologies are integrated into routine clinical practice [15].

Survey-based evidence from other health care systems supports our observation. A German national survey demonstrated that clinicians working in academic practices were significantly more likely to use prostate MRI and fusion biopsy than those in community settings, largely reflecting differences in access to specialized imaging expertise and equipment [18]. Similar adoption patterns have been reported across multiple surgical domains, including minimally invasive colorectal surgery, endovascular aneurysm repair, thoracic surgery, and robot-assisted prostatectomy, with academic centers consistently acting as early adopters of guideline-endorsed techniques [15]. Despite overall implementation, adoption remained incomplete in nonuniversity settings, with a small but persistent use of non–MRI-guided biopsy even in recent years. Current guidelines allow direct biopsy without prior MRI in selected high-risk scenarios, such as patients with clinically advanced disease (cT3–4) or markedly elevated PSA levels (eg, >50 ng/ml). However, such cases are expected to be rare in a prostatectomy-based registry, as these patients are typically not managed with primary surgical treatment [2]. This is supported by our data, in which only 40 patients (0.5%) had these criteria. Although, in theory, such a selection effect, where patients with higher-risk disease may undergo systematic biopsy and patients with lower-risk disease are more likely to receive MRI, could influence biopsy patterns, its impact on our findings is likely limited given the very small proportion of these cases. At the same time, it should be noted that even in patients with high-risk disease, preoperative MRI may still provide important clinical value, particularly for local staging, surgical planning, and assessment of resectability before radical prostatectomy. Regional and private hospitals demonstrated very similar adoption patterns, reinforcing our hypothesis that institutional setting, rather than ownership alone, shapes implementation dynamics. These findings suggest that factors beyond access and cost, including organizational culture, established workflows, and clinician behavior, influence the uptake of guideline-recommended innovations.

Beyond adoption patterns, the multivariable analyses provide evidence that biopsy technique is not merely a marker of institutional practice but is independently associated with clinically meaningful downstream outcomes. After comprehensive adjustment for patient, tumor, temporal, and institutional covariates, MRI-guided biopsy was associated with a significantly lower risk of clinical understaging and PSM, suggesting improved alignment between preoperative assessment and definitive pathology. mpMRI improves visualization of tumor extent and localization, particularly for anterior, apical, and small-volume clinically significant lesions that are frequently underdetected by systematic transrectal ultrasound (TRUS) biopsy; accordingly, the observed reduction in clinical understaging is not attributable to the biopsy technique itself, as staging definitions are not influenced by the biopsy procedure, but rather reflects the use of prebiopsy MRI, which enables more accurate staging. By enabling targeted sampling of the radiologically dominant lesion, MRI-guided biopsy reduces the likelihood that relevant tumor burden is missed preoperatively, thereby improving alignment between clinical staging and definitive pathology [7], [8], [9]. Improved preoperative spatial characterization also explains the observed association with more favorable margin status. Accurate localization of tumor laterality and extent allows surgeons to tailor resection plans more precisely and balance oncological control with functional preservation. This interpretation is consistent with previous studies showing that MRI-informed diagnostic pathways can improve surgical planning and pathological outcomes, without increasing operative risk [7], [8]. In contrast, MRI-guided biopsy was not associated with differences in ISUP grade upgrading at prostatectomy, reflecting persistent limitations of PCa diagnostics because of tumor multifocality, biological heterogeneity, and residual biopsy sampling inaccuracy. Consistent with randomized trials, this suggests that the primary value of MRI-guided biopsy lies in improved spatial characterization and staging accuracy rather than in eliminating grade discordance [8], [10]. Although MRI-guided biopsy improves detection of clinically significant disease, it may also lead to apparent upgrading at biopsy level compared to systematic biopsy, with subsequent downgrading at prostatectomy, potentially affecting eligibility for active surveillance, as demonstrated by Ahdoot et al [19]. In their study, downgrading to clinically insignificant disease after combined biopsy was observed in approximately 3.7% of the cases, a relatively infrequent but clinically relevant phenomenon, as it may lead to exclusion from active surveillance in patients who ultimately harbor low-risk disease. Notably, biopsy year was the strongest predictor of reduced upgrading, likely reflecting maturation of diagnostic pathways, including increased MRI availability, radiological expertise, and standardized reporting, underscoring the importance of experience and infrastructure. Together, these findings highlight that MRI-guided biopsy not only improves diagnostic accuracy but also enhances preoperative planning, with downstream benefits for surgical outcomes, and should therefore be interpreted as part of an MRI-informed diagnostic pathway rather than as an isolated procedural effect.

The Swiss experience likely represents a best-case scenario rather than typical real-world conditions. Switzerland benefits from high MRI density, universal insurance coverage, strong subspecialization, and supportive financing structures that facilitate rapid technological uptake. In other health care systems, access to MRI may be limited, radiology workforce shortages may constrain capacity, and reimbursement structures may pose additional barriers to implementation [18]. Consequently, the adoption differences observed within Switzerland likely overestimate the magnitude of disparities in other countries.

This study has several limitations. First, the analysis was restricted to patients undergoing radical prostatectomy, which limits generalizability to men managed with active surveillance, radiotherapy, or focal therapy, but provides the methodological advantage of definitive pathological reference. Second, although the analysis was based on prospectively collected data, its observational design precludes causal inference, and residual confounding cannot be excluded. Although models were adjusted for hospital type as a proxy for institutional differences, more granular physician- and center-level characteristics (eg, surgeon experience, center volume, or radiological expertise) were not available in the registry and could not be included. Clustering at the individual center level was not accounted for, and the biopsy technique may partly reflect unmeasured differences in provider or institutional quality, thereby acting as a proxy for these factors rather than representing an isolated effect of the diagnostic approach itself. Third, neither MRI quality nor reporting expertise were formally assessed using a dedicated quality metric such as PI-QUAL nor was reader experience systematically captured, which may have introduced heterogeneity in MRI performance across centers [20]. Fourth, long-term oncological outcomes, like biochemical recurrence, were not assessed because of the relatively short follow-up of this contemporary cohort; however, this represents an important focus for future analyses as longer-term registry data become available. Fifth, information on staging modalities, such as prostate-specific membrane antigen positron emission tomography imaging, were not captured in the registry. Therefore, the observed associations should be interpreted with caution, as they may partly reflect temporal changes in diagnostic pathways rather than a direct effect of the biopsy technique alone. Finally, differentiation between MRI-targeting techniques was not possible, and hospital-level factors, such as organizational structure and radiology resources, could not be directly assessed.

In summary, MRI-guided biopsy has rapidly become the dominant diagnostic approach in Switzerland, with university hospitals acting as early adopters and clear drivers of practice change, followed by a gradual convergence across nonuniversity care settings. Using high-quality data from a large, prospective national registry characterized by minimal missing data, this study captures 5-yr adoption trends during a period of evolving guideline recommendations and underscores the pivotal role of academic institutions as trendsetters for innovative, evidence-based diagnostic strategies. By implementing MRI-guided biopsy earlier and more comprehensively, university centers likely accelerated nationwide diffusion, facilitated experiential learning, and set benchmarks for diagnostic and surgical quality. Importantly, this timely dissemination of innovation was associated with improved staging accuracy and surgical quality without increasing perioperative risk, highlighting how academic leadership can translate advances in diagnostic technology into measurable benefits across an entire health care system.

## 5 Conclusions

In this nationwide analysis, MRI-guided biopsy rapidly supplanted non–MRI-guided techniques, with university hospitals acting as early adopters and other care settings converging thereafter. After multivariable adjustment, MRI-guided biopsy was associated with reduced risk of clinical understaging and PSM; however, these observed differences should be interpreted with caution, as they may reflect differences in institutional expertise and care pathways rather than the biopsy technique itself. These findings highlight the central role of academic centers in driving the adoption of evidence-based technologies, generating benefits that extend beyond individual patients to the health care system as a whole through downstream diffusion and standardization of care.

  ***Author contributions:*** Nicola Giudici had full access to all the data in the study and takes responsibility for the integrity of the data and the accuracy of the data analysis.

  *Study concept and design*: Giudici, Roth.

*Acquisition of data*: Wyler, Nguyen, Abt, Mattei, Strebel, Roth.

*Analysis and interpretation of data*: Giudici, Tasios, Roth.

*Drafting of the manuscript*: Giudici, Tasios.

*Critical revision of the manuscript for important intellectual content*: Arnold, Röthlisberger, Thalmann, Schneidewind, Roth.

*Statistical analysis*: Giudici.

*Obtaining funding*: None.

*Administrative, technical, or material support*: None.

*Supervision*: Roth, Giudici.

*Other* (specify): None.

  ***Financial disclosures:*** Nicola Giudici certifies that all conflicts of interest, including specific financial interests and relationships and affiliations relevant to the subject matter or materials discussed in the manuscript (eg, employment/affiliation, grants or funding, consultancies, honoraria, stock ownership or options, expert testimony, royalties, or patents filed, received, or pending), are the following: None.

  ***Funding/Support and role of the sponsor:*** None.

  ***Ethics approval:*** The protocol was approved by the Cantonal Ethics Committee of Zurich (BASEC-Nr. Req-2017-00283).

  ***Availability of data and materials:*** Data cannot be shared for ethical/privacy reasons. Requests for access to deidentified data may be considered upon reasonable request to the corresponding author and subject to approval by the Swiss Urology Registry governance and applicable data protection regulations.

## Appendix A Supplementary data

Supplementary Appendix Supplementary data contains additional tables, collaborator list, and multivariable regression analyses.
