# Supplementary material for: Adoption of MRI-guided Prostate Cancer Diagnostics and Surgical Outcomes: A Prospective Multicenter Registry Study
Source: Eur Urol Open Sci. 2026 Jul 10;90:121–8. doi: 10.1016/j.euros.2026.05.021 (PMC13377140; doi:10.1016/j.euros.2026.05.021)
Supplement: Supplementary Appendix — Supplementary data contains additional tables, collaborator list, and multivariable regression analyses. [file mmc1.docx]

**Supplementary Appendix**

**Adoption of MRI-Guided Prostate Cancer Diagnostics and Surgical Outcomes: A Prospective Multicenter Registry Study**

**Table 1. Collaborators of the Swiss Urology Registry**

| **Investigator** | **Center (Switzerland)** |
| --- | --- |
| Dr. Karim Kellou | Hôpital de Nyon, Nyon, Switzerland |
| Dr. med. Thomas Sautter | Uroclinic, Wetzikon, Switzerland |
| Dr. med. Wolfgang Schäfer | Spitalzentrum Oberwallis, Visp, Switzerland |
| Anja Rieger | Limmattalspital, Schlieren, Switzerland |
| Dr. med. Simone Brunschweiler | Bellevue Urology, Zürich, Switzerland |
| Dr. Thomas Tawadros | Hôpital Riviera-Chablais, Rennaz, Switzerland |
| Dr. med. Thomas Sautter | Uroclinic, Pfäffikon, Switzerland |
| Dr. Aron Cohen | Urocare AG, Küsnacht, Switzerland |
| PD Dr. méd. Daniel Nguyen | Hôpital Neuchâtelois, Neuchâtel, Switzerland |
| Dr. med. Gautier Müllhaupt | Spital Thun (SpitalSTS AG), Thun, Switzerland |
| Dr. med. Stefan Preusser | Kantonsspital Schaffhausen, Schaffhausen, Switzerland |
| Prof. Ilaria Lucca | CHUV - Centre Hospitalier Universitaire Vaudois, Lausanne, Switzerland |
| Prof. Beat Roth | Inselspital, Bern, Switzerland |
| Dr. Thomas Luginbühl | Spital Uster, Uster, Switzerland |
| Dr. med. Roland Seiler | Spitalzentrum Biel, Biel, Switzerland |
| Astrid Bergundthal | Hirslanden Klinik Stephanshorn, St. Gallen, Switzerland |
| Olivier Ischer & Jérôme Chaptinel | Clinique de la Source, Lausanne, Switzerland |
| Prof. Massimo Valerio | Hôpitaux Universitaires de Genève (HUG), Genève, Switzerland |
| PD Dr. med. Räto Strebel | Kantonsspital Graubünden, Chur, Switzerland |
| PD Dr. Daniel Engeler | Kantonsspital St. Gallen (KSSG), St. Gallen, Switzerland |
| Prof. Daniel Eberli | Universitätsspital Zürich (USZ), Zürich, Switzerland |
| PD Dr. med. Tobias Zellweger | St. Claraspital, Basel, Switzerland |
| Prof. Dr. med. Agostino Mattei | Luzerner Kantonsspital (LUKS), Luzern, Switzerland |
| Prof. Dr. med. Hubert John | Kantonsspital Winterthur (KSW), Winterthur, Switzerland |
| Prof. Dr. med. Stephen Wyler | Kantonsspital Aarau, Aarau, Switzerland |
| Denise Bundi | Uroviva, Bülach, Switzerland |
| Julien Schwartz | Hirslanden Clinique Cecil, Lausanne, Switzerland |
| Dr. med. Stephan Bauer | Hirslanden Zentrum für Urologie (ZfU), Zürich, Switzerland |

**Table 2. Baseline, Operative, and Pathologic Characteristics of TURP / Other Diagnostic Pathway**

| **Variable** | **TURP / Other (N = 356)** |
| --- | --- |
| Institution & demographics |  |
| University hospital, n (%) | 24 (6.7%) |
| Regional hospital, n (%) | 94 (26.4%) |
| Local / private hospital, n (%) | 238 (66.9%) |
| Age, years, median [IQR] | 67.0 [62-72] |
| Body mass index, kg/m², median [IQR] | 26.0 [24.1-28.1] |
| ASA I, n (%) | 16 (4.5%) |
| ASA II, n (%) | 173 (48.6%) |
| ASA III, n (%) | 165 (46.3%) |
| **Preoperative characteristics** |  |
| Preoperative PSA, ng/mL, median [IQR] | 5.8 [4.1-8.6] |
| D’Amico low-risk, n (%) | 25 (7.0%) |
| D’Amico intermediate-risk, n (%) | 226 (63.5%) |
| D’Amico high-risk, n (%) | 98 (27.5%) |
| Prostate volume, mL, median [IQR] | 40.0 [29-50.5] |
| Number of biopsy cores taken, median [IQR] | 15 [12-18] |
| Number of positive biopsy cores, median [IQR] | 5 [2-8] |
| **Operative characteristics** |  |
| Robotic/laparoscopic-assisted approach, n (%) | 323 (90.7%) |
| Open approach, n (%) | 33 (9.3%) |
| Pelvic lymph-node dissection, n (%) | 297 (83.4%) |
| Nerve-sparing performed, n (%) | 87 (24.4%) |
| Estimated blood loss, mL, median [IQR] | 300 [200-400] |
| Operative time, min, median [IQR] | 240 [205-280] |
| **Postoperative and pathologic outcomes** |  |
| Pathologic T stage pT2, n (%) | 251 (70.5%) |
| Pathologic T stage ≥pT3, n (%) | 100 (28.1%) |
| Postoperative ISUP grade 1-2, n (%) | 223 (62.6%) |
| Postoperative ISUP grade 3-5, n (%) | 130 (36.5%) |
| Positive surgical margin, n (%) | 57 (16.0%) |
| Lymph nodes examined, n, median [IQR] | 10 [5-16] |
| Any intraoperative complication, n (%) | 6 (1.7%) |
| Any postoperative complication (30-day), n (%) | 38 (10.7%) |
| Clavien-Dindo I-II, n (%) | 19 (5.3%) |
| Clavien-Dindo ≥ III, n (%) | 7 (2.0%) |

**Table 3. Trends in Biopsy Technique Use (2020-2025) by Hospital Type**

|  | **2020H1** | **2020H2** | **2021H1** | **2021H2** | **2022H1** | **2022H2** | **2023H1** | **2023H2** | **2024H1** | **2024H2** | **2025H1** |
| --- | --- | --- | --- | --- | --- | --- | --- | --- | --- | --- | --- |
| **Academic - TRUS** | 1/27 (3.7%) | 5/47 (10.6%) | 4/48 (8.3%) | 4/63 (6.3%) | 8/91 (8.8%) | 1/75 (1.3%) | 1/104 (1.0%) | 1/93 (1.1%) | 1/95 (1.1%) | 0/60 (0.0%) | 0/1 (0.0%) |
| **Academic - MRI-guided** | 23/27 (85.2%) | 40/47 (85.1%) | 44/48 (91.7%) | 57/63 (90.5%) | 82/91 (90.1%) | 74/75 (98.7%) | 99/104 (95.2%) | 85/93 (91.4%) | 89/95 (93.7%) | 60/60 (100.0%) | 1/1 (100.0%) |
| **Academic - TUR-P/Other** | 3/27 (11.1%) | 2/47 (4.3%) | 0/48 (0.0%) | 2/63 (3.2%) | 1/91 (1.1%) | 0/75 (0.0%) | 4/104 (3.8%) | 7/93 (7.5%) | 5/95 (5.3%) | 0/60 (0.0%) | 0/1 (0.0%) |
| **Regional hospital - TRUS** | 44/87 (50.6%) | 44/96 (45.8%) | 69/197 (35.0%) | 66/272 (24.3%) | 108/363 (29.8%) | 64/289 (22.1%) | 60/318 (18.9%) | 48/280 (17.1%) | 66/327 (20.2%) | 34/214 (15.9%) | 2/21 (9.5%) |
| **Regional hospital - MRI-guided** | 40/87 (46.0%) | 46/96 (47.9%) | 113/197 (57.4%) | 191/272 (70.2%) | 242/363 (66.7%) | 208/289 (72.0%) | 248/318 (78.0%) | 225/280 (80.4%) | 258/327 (78.9%) | 177/214 (82.7%) | 19/21 (90.5%) |
| **Regional hospital - TUR-P/Other** | 3/87 (3.4%) | 6/96 (6.2%) | 15/197 (7.6%) | 15/272 (5.5%) | 13/363 (3.6%) | 17/289 (5.9%) | 10/318 (3.1%) | 7/280 (2.5%) | 3/327 (0.9%) | 3/214 (1.4%) | 0/21 (0.0%) |
| **Local/private - TRUS** | 79/144 (54.9%) | 106/287 (36.9%) | 168/529 (31.8%) | 147/505 (29.1%) | 151/547 (27.6%) | 130/489 (26.6%) | 99/489 (20.2%) | 104/450 (23.1%) | 93/491 (18.9%) | 35/305 (11.5%) | 3/21 (14.3%) |
| **Local/private - MRI-guided** | 62/144 (43.1%) | 166/287 (57.8%) | 339/529 (64.1%) | 334/505 (66.1%) | 373/547 (68.2%) | 330/489 (67.5%) | 358/489 (73.2%) | 313/450 (69.6%) | 371/491 (75.6%) | 250/305 (82.0%) | 18/21 (85.7%) |
| **Local/private  - TUR-P/Other** | 3/144 (2.1%) | 15/287 (5.2%) | 22/529 (4.2%) | 24/505 (4.8%) | 23/547 (4.2%) | 29/489 (5.9%) | 32/489 (6.5%) | 33/450 (7.3%) | 27/491 (5.5%) | 20/305 (6.6%) | 0/21 (0.0%) |

**Abbreviations:** TRUS = transrectal ultrasound

**Table 4. Multivariable Logistic Regression for Predictors of ISUP Upgrading After Radical Prostatectomy**

| **Variable** | **Adjusted OR** | **95% CI** | **p-value** |
| --- | --- | --- | --- |
| **Biopsy technique** |  |  |  |
| Non- MRI-guided (Ref) | - | - | - |
| MRI-guided | 1.10 | 0.95 - 1.27 | 0.200 |
| **Age (years)** | 1.01 | 1.00 - 1.02 | 0.085 |
| **Preoperative PSA** | 1.00 | 1.00 - 1.00 | 0.108 |
| **cT stage** |  |  |  |
| cT1 (Ref) | - | - | - |
| cT2 | 0.97 | 0.85 - 1.12 | 0.707 |
| cT3-4 | 0.99 | 0.83 - 1.18 | 0.899 |
| **Prostate volume (mL)** | 1.00 | 1.00 - 1.00 | 0.695 |
| **Hospital type** |  |  |  |
| University (Ref) | - | - | - |
| Other | 1.14 | 0.93 - 1.39 | 0.224 |
| **Year of biopsy** | 0.91 | 0.88 - 0.95 | <0.001 |
| **ASA score** |  |  |  |
| ASA I (Ref) | - | - | - |
| ASA II | 1.18 | 0.92 - 1.52 | 0.186 |
| ASA III | 1.17 | 0.90 - 1.53 | 0.244 |
| **Surgical technique** |  |  |  |
| Open (Ref) | - | - | - |
| Robotic | 1.02 | 0.82 - 1.27 | 0.871 |

**Table 5. Multivariable Logistic Regression for Predictors of Understaging (cT < pT)**

| **Variable** | **Adjusted OR** | **95% CI** | **p-value** |
| --- | --- | --- | --- |
| **Biopsy technique** |  |  |  |
| Non- MRI-guided (Ref) | - | - | - |
| MRI-guided | 0.64 | 0.57 - 0.72 | <0.001 |
| **Age (years)** | 1.00 | 0.99 - 1.00 | 0.387 |
| **Preoperative PSA** | 1.00 | 1.00 - 1.00 | 0.669 |
| **ISUP grade (preoperative)** |  |  |  |
| ISUP 1 (Ref) | - | - | - |
| ISUP 2 | 1.06 | 0.91 - 1.23 | 0.483 |
| ISUP 3 | 0.98 | 0.82 - 1.17 | 0.830 |
| ISUP 4 | 0.68 | 0.57 - 0.82 | <0.001 |
| ISUP 5 | 0.83 | 0.63 - 1.08 | 0.170 |
| **Prostate volume (mL)** | 1.00 | 1.00 - 1.00 | 0.419 |
| **Hospital type** |  |  |  |
| University (Ref) | - | - | - |
| Other hospitals | 0.34 | 0.28 - 0.41 | <0.001 |
| **Year of biopsy** | 1.13 | 1.08 - 1.17 | <0.001 |
| **ASA score** |  |  |  |
| ASA I (Ref) | - | - | - |
| ASA II | 0.81 | 0.66 - 1.00 | 0.049 |
| ASA III | 1.01 | 0.81 - 1.26 | 0.959 |
| **Surgical technique** |  |  |  |
| Open (Ref) | - | - | - |
| Robotic | 0.94 | 0.78 - 1.13 | 0.504 |

**Table 6. Multivariable Logistic Regression for Predictors of Positive Surgical Margins (PSM)**

| **Variable** | **Adjusted OR** | **95% CI** | **p-value** |
| --- | --- | --- | --- |
| **Biopsy technique** |  |  |  |
| Non- MRI-guided (Ref) | - | - | - |
| MRI-guided | 0.80 | 0.70 - 0.92 | 0.0013 |
| **Age (years)** | 1.01 | 1.00 - 1.01 | 0.249 |
| **Preoperative PSA (ng/mL)** | 1.02 | 1.02 - 1.03 | <0.001 |
| **ISUP preoperative** |  |  |  |
| ISUP 1 (Ref) | - | - | - |
| ISUP 2 | 1.12 | 0.93 - 1.34 | 0.250 |
| ISUP 3 | 1.28 | 1.04 - 1.58 | 0.019 |
| ISUP 4 | 1.19 | 0.95 - 1.49 | 0.121 |
| ISUP 5 | 2.29 | 1.71 - 3.06 | <0.001 |
| **Clinical T-stage** |  |  |  |
| T1 (Ref) | - | - | - |
| T2 | 0.80 | 0.70 - 0.92 | 0.0013 |
| T3-4 | 1.18 | 1.00 - 1.40 | 0.050 |
| **Prostate volume (mL)** | 0.99 | 0.99 - 0.99 | <0.001 |
| **Hospital type** |  |  |  |
| University (Ref) | - | - | - |
| Other hospital | 1.37 | 1.11 - 1.69 | 0.0036 |
| **Year of biopsy** | 1.02 | 0.98 - 1.06 | 0.415 |
| **ASA score** |  |  |  |
| ASA I (Ref) | - | - | - |
| ASA II | 1.17 | 0.92 - 1.50 | 0.206 |
| ASA III | 1.23 | 0.94 - 1.59 | 0.127 |
| **Surgical technique** |  |  |  |
| Open (Ref) | - | - | - |
| Robotic | 0.77 | 0.63 - 0.94 | 0.012 |

**Table 7. Multivariable Logistic Regression for Predictors of Overall Complications**

| **Variable** | **Adjusted OR** | **95% CI** | **p-value** |
| --- | --- | --- | --- |
| **Biopsy technique** |  |  |  |
| Non- MRI-guided (Ref) | - | - | - |
| MRI-guided | 1.03 | 0.84 - 1.27 | 0.764 |
| **Age (years)** | 1.01 | 0.99 - 1.02 | 0.317 |
| **Preoperative PSA (ng/mL)** | 1.00 | 1.00 - 1.00 | 0.074 |
| **ISUP preoperative** |  |  |  |
| ISUP 1 (Ref) | - | - | - |
| ISUP 2 | 1.22 | 0.92 - 1.60 | 0.163 |
| ISUP 3 | 1.34 | 0.98 - 1.81 | 0.063 |
| ISUP 4 | 1.37 | 1.00 - 1.89 | 0.050 |
| ISUP 5 | 1.96 | 1.31 - 2.94 | 0.001 |
| **Clinical T stage** |  |  |  |
| cT1 (Ref) | - | - | - |
| cT2 | 0.92 | 0.76 - 1.11 | 0.395 |
| cT3-4 | 0.94 | 0.73 - 1.21 | 0.643 |
| **Prostate volume (mL)** | 1.00 | 1.00 - 1.00 | 0.649 |
| **Hospital type** |  |  |  |
| University (Ref) | - | - | - |
| Other | 0.47 | 0.37 - 0.60 | <0.001 |
| **Year of biopsy** | 0.96 | 0.92 - 1.01 | 0.090 |
| **ASA score** |  |  |  |
| ASA I (Ref) | - | - | - |
| ASA II | 1.01 | 0.71 - 1.43 | 0.975 |
| ASA III | 1.24 | 0.86 - 1.80 | 0.247 |
| **Surgical technique** |  |  |  |
| Open surgery (Ref) | - | - | - |
| Robotic | 0.44 | 0.34 - 0.56 | <0.001 |
